# SiLEA14, a novel atypical LEA protein, confers abiotic stress resistance in foxtail millet

**DOI:** 10.1186/s12870-014-0290-7

**Published:** 2014-11-18

**Authors:** Meizhen Wang, Ping Li, Cong Li, Yanlin Pan, Xiyuan Jiang, Dengyun Zhu, Qian Zhao, Jingjuan Yu

**Affiliations:** State Key Laboratory of Agrobiotechnology, College of Biological Sciences, China Agricultural University, No. 2 Yuanmingyuan West Road, Haidian District, Beijing, 100193 China; Institute of Medicinal Plant Development, Chinese Academy of Medical Sciences & Peking Union Medical College, No. 151, Malianwa North Road, Haidian District, Beijing, 100193 China

## Abstract

**Background:**

Late embryogenesis abundant (LEA) proteins are involved in protecting higher plants from damage caused by environmental stresses. Foxtail millet (*Setaria italica*) is an important cereal crop for food and feed in semi-arid areas. However, the molecular mechanisms underlying tolerance to these conditions are not well defined.

**Results:**

Here, we characterized a novel atypical LEA gene named *SiLEA14* from foxtail millet. It contains two exons separated by one intron. *SiLEA14* was expressed in roots, stems, leaves, inflorescences and seeds at different levels under normal growth conditions. In addition, *SiLEA14* was dramatically induced by osmotic stress, NaCl and exogenous abscisic acid. The SiLEA14 protein was localized in the nucleus and the cytoplasm. Overexpression of *SiLEA14* improved *Escherichia coli* growth performance compared with the control under salt stress. To further assess the function of *SiLEA14* in plants, transgenic *Arabidopsis* and foxtail millet plants that overexpressed *SiLEA14* were obtained. The transgenic *Arabidopsis* seedlings showed higher tolerance to salt and osmotic stress than the wild type (WT). Similarly, the transgenic foxtail millet showed improved growth under salt and drought stresses compared with the WT. Taken together, our results indicated that SiLEA14 is a novel atypical LEA protein and plays important roles in resistance to abiotic stresses in plants.

**Conclusion:**

We characterized a novel atypical LEA gene *SiLEA14* from foxtail millet, which plays important roles in plant abiotic stress resistance. Modification of *SiLEA14* expression may improve abiotic stress resistance in agricultural crops.

**Electronic supplementary material:**

The online version of this article (doi:10.1186/s12870-014-0290-7) contains supplementary material, which is available to authorized users.

## Background

Environmental stresses, such as drought and high salinity, can cause severe damage to plants, leading to considerable reduction in their productivity. To survive under such conditions, plants have developed a series of defense responsive pathways. Among them, Ca^2+^-dependent signaling leads to the activation of late embryogenesis abundant (LEA)-type genes, which may function in protection and damage repair of plants [1].

LEA proteins were first identified in cotton seeds [2]. The proteins accumulated to high levels in the late stages of seed development [3,4]. Subsequently, they were found to be expressed in vegetative tissues [5,6] and could be induced by abscisic acid (ABA) and various abiotic stresses, such as drought and cold [5-9]. With the development of deep sequencing technology, an increasing number of LEA proteins have been identified. On the basis of their amino acid sequence similarities and conserved motifs, LEA proteins are categorized into different groups [10-13]. In this work, we adopt the classification introduced by Battaglia’s group, in which LEA proteins are categorized into seven distinct families [12]. Groups 1, 2, 3, 4, 6 and 7, which share specific motifs within each group, are considered to be hydrophilic or “typical” LEA proteins. Conversely, group 5 corresponds to atypical LEA proteins. This group includes all LEA proteins with higher content of hydrophobic residues than typical LEA proteins. On the basis of their sequence similarity, group 5 LEA proteins are divided into the subgroups 5A, 5B, and 5C, corresponding to the first described proteins D-34, D-73, and D-95 [3,14] in this group, respectively. Physicochemical properties show that group 5 LEA proteins are not soluble after boiling, suggesting that they may adopt a globular conformation and are not heat stable [3,15,16]. Subsequent reports show that subgroup 5C LEA proteins are natively folded and have more β-sheets than α-helixes [5,17], which is different from subgroups 5A and 5B LEA proteins that are intrinsically unstructured [17-19]. For example, Arabidopsis LEA14 has an αβ-fold consisting of one α-helix and seven β-strands that form two antiparallel β-sheets as determined by nuclear magnetic resonance spectroscopy [16]. Moreover, subgroup 5C LEA proteins have other outstanding characteristics, such as lower instability index, narrower range of GRAVY values, and lower proportion of polar (hydrophilic) and small residues, but higher proportion of non-polar residues than subgroup 5A and 5B members [5]. All of these differences indicate that subgroup 5C LEA proteins may function differently from other LEA proteins in plants.

At present, only a small number of subgroup 5C LEA genes have been characterized. Their transcripts can be upregulated in response to diverse stresses, as reported for cotton *LEA14-A* [15], *Craterostigma plantagineum PcC27-45* [20], soybean *D95-4* [21], tomato *ER5* [22], hot pepper *CaLEA6* [23], Arabidopsis *LEA14* [24] and *At2g44060* [17], and sweetpotato *IbLEA14* [25]. Overexpression of *CaLEA6* in tobacco improves tolerance to dehydration and NaCl but not to low temperature [23]. Transgenic sweetpotato non-embryogenic calli that overexpress *IbLEA14* show increased tolerance to drought and salt stress by enhancing lignification [25]. Recently, rice *OsLEA5* has been reported to enhance resistance against diverse abiotic stresses in recombinant *Escherichia coli* cells. In vitro analysis showed that OsLEA5 was able to protect lactate dehydrogenase from aggregation under different abiotic stresses [5]. All these results suggest that subgroup 5C LEA proteins are closely associated with resistance to multiple abiotic stresses.

Foxtail millet (*Setaria italica* (L.) Beauv.), a member of the Poaceae family, has a long history in cultivation of about 7000 years. It has been widely planted in northern China and other Asian countries. Recently, a draft genome sequence for foxtail millet has been completed [26], which enables foxtail millet to be a tractable experimental grass model [27]. As a diploid C_4_ panicoid crop species, foxtail millet is well known for its remarkable drought resistance. However, the molecular mechanisms underlying this tolerance are not well defined.

In this study, we isolated and functionally characterized a novel member of the atypical subgroup 5C LEA gene, *SiLEA14*, from foxtail millet. The expression of *SiLEA14* was induced by ABA, polyethylene glycol (PEG) and NaCl. Overexpression of *SiLEA14* resulted in enhanced resistance to abiotic stresses in *E. coli*, Arabidopsis and foxtail millet. The *SiLEA14* promoter mediated remarkable induction of β-glucuronidase (GUS) expression in transgenic Arabidopsis under various stresses. *Cis*-acting regulatory elements in the *SiLEA14* promoter were also predicted. These data reveal the potential application of *SiLEA14* in the genetic engineering of other crops.

## Results

### SiLEA14 is an atypical LEA protein

The full-length sequence of *SiLEA14* was determined by 5′ and 3′ rapid amplification of cDNA ends (RACE) [GenBank: KJ767551]. The sequence is 821 bp in length, with a 100 bp 5′ untranslated region (UTR) and a 208 bp 3′ UTR (with polyA tail) (Additional file 1A). *SiLEA14* harbors two exons separated by an intron (Additional file 1B), and encodes an open reading frame of 170 aa with a predicted molecular mass of 18.77 kD and pI of 5.56. It is rich in Ser (10.6%), Lys (8.8%), and Ile (8.2%), but contains low quantities of Trp (1.2%), Asn (1.8%), Cys (1.8%), and Gln (1.8%). Three hydrophobic regions (I, II, and III) were identified in the SiLEA14 protein (Additional file 1C). A motif search of the SiLEA14 protein in InterProScan revealed that it contains a “LEA_2” motif (PF03186, 3.4e-20), which was classified into subgroup 5C (D-95) according to Battaglia’s classification of LEA proteins [12]. Further analysis showed that SiLEA14 contains a lower percentage of polar amino acids (47.2%) and higher percentage of non-polar amino acids (25.8%) than other group LEA proteins (Additional file 1D) [5]. The GRAVY value and the instability index of SiLEA14 is −0.155 and 34.68, respectively (Additional file 1D). All these characteristics were in accordance with those of other members of subgroup 5C [5].

**Figure 1:**
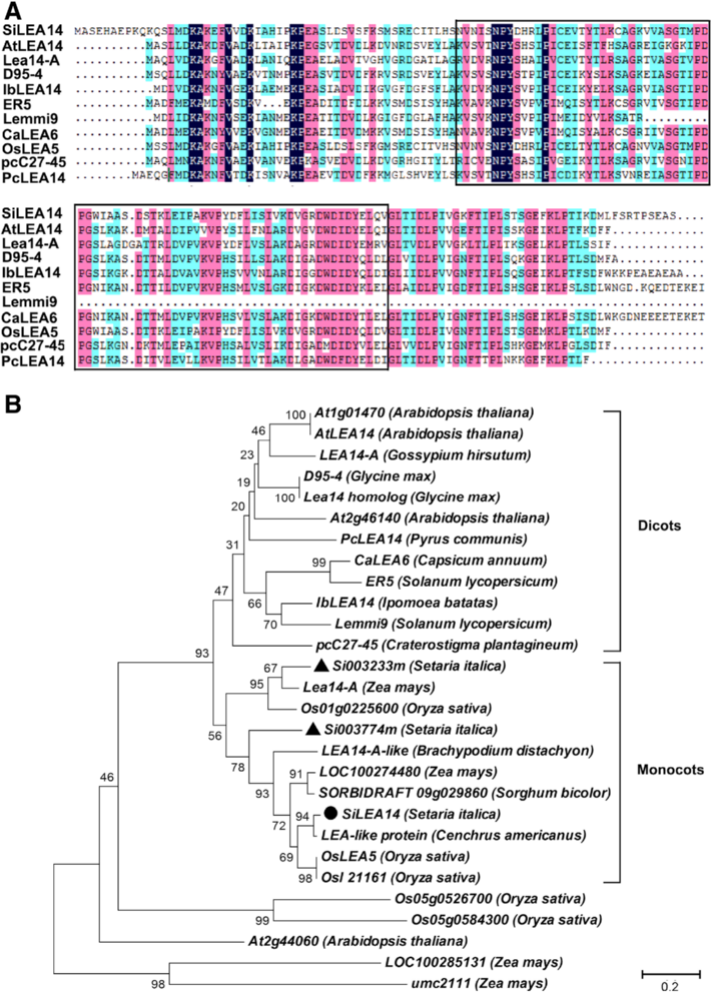
**Multiple sequence alignment and phylogenetic relationship for the SiLEA14 protein and its homologs. (A)** Multiple sequence alignment of *SiLEA14* with its homologs from various plant species. The conserved “LEA_2” motif (PF03168) is boxed. **(B)** Phylogenetic relationships of SiLEA14 and its homologs. The divergence of the clades between the monocots and dicots is highlighted. SiLEA14 and its two homologs in foxtail millet are highlighted by solid dark circle and triangle, respectively. The GenBank accession numbers are as follows: SiLEA14 (*Setaria italic*, KJ767551), AtLEA14 (*Arabidopsis thaliana*, NM_100029), Lea14-A (*Zea mays*, NM_001159174), D95-4 (*Glycine max*, U08108), IbLEA14 (*Ipomoea batatas*, GU369820), ER5 (*Solanum lycopersicum*, U77719), Lemmi9 (*Solanum lycopersicum*, Z46654), CaLEA6 (*Capsicum annuum*, AF168168), OsLEA5 (*Oryza sativa*, JF776156), pcC27-45 (*Craterostigma plantagineum*, M62990), pcLEA14 (*Pyrus communis*, AF386513), At1g01470 (*Arabidopsis thaliana*, BT015111), LEA14-A (*Gossypium hirsutum*, M88322), Lea14 homolog (*Glycine max*, NM_001251780), At2g46140 (*Arabidopsis thaliana*, NM_130176), Os01g0225600 (*Oryza sativa*, NM_001048996), LEA14-A-like (*Brachypodium distachyon*, XM_003567779), LOC100274480 (*Zea mays*, NM_001148839), SORBIDRAFT 09 g029860 (*Sorghum bicolour*, XM_002441543), LEA-like protein (*Cenchrus americanus*, AY823547), OsI21161 (*Oryza sativa*, CM000130), Os05g0526700 (*Oryza sativa*, NM_001062639), Os05g0584300 (*Oryza sativa*, NM_001062985), At2g44060 (*Arabidopsis thaliana*, BT024723), LOC100285131 (*Zea mays*, EU970969) and umc2111 (*Zea mays*, NM_001155750). The sequences used for alignment and phylogenetic tree construction were shown in Additional file 2.

Two additional SiLEA14 homologs (Si003774m and Si003233m, Additional file 2) existed in foxtail millet. They showed 57.7% and 48.24% identity to SiLEA14, respectively. Homologous sequences of SiLEA14 were also present in other plant species, such as rice OsLEA5 (77.06%), maize Lea14-A (50.88%) and Arabidopsis AtLEA14 (47.06%). The homologs shared different levels of sequence similarities, indicating different levels of evolutionary relationship among these proteins (Figure 1A). A phylogenetic tree for SiLEA14 and its homologs was constructed from protein sequences (Additional file 2). The proteins were mainly separated into two clades comprising dicot and monocot species, respectively. The SiLEA14 protein shared the closest relationship with the pearl millet LEA-like protein, followed by OsLEA5 (Figure 1B). Sequence logos were produced to examine the conservation level at each residue position. The sequences lacked significant signature motifs or consensus sequences, except for several conserved residues at specific positions, such as proline at positions 41 and 83 (Additional file 3). This is a common feature of subgroup 5C LEA proteins [5].

### Subcellular localization of SiLEA14

**Figure 2:**
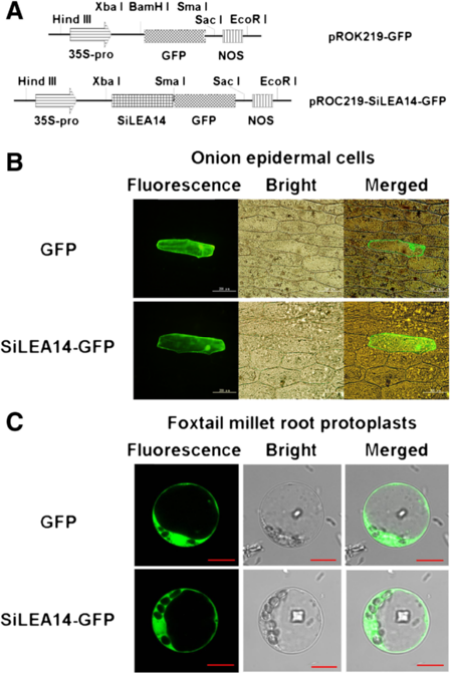
**Subcellular localization of SiLEA14 protein. (A)** Diagram of the constructs of pROK219-GFP and pROK219-SiLEA14-GFP used for subcellular localization. 35S-Pro, *cauliflower mosaic virus* 35S promoter; SiLEA14, the SiLEA14 coding region without the stop codon; GFP, green fluorescent protein; NOS, nopaline synthase terminator. **(B-C)** Fluorescent microscopic images of GFP and SiLEA14-GFP fusion protein in the onion epidermal cells (Bar = 200 μm) **(B)** and in the foxtail millet root protoplasts (Bar = 10 μm) **(C)**.

To investigate the potential role of SiLEA14, we examined the subcellular localization of SiLEA14 fused to GFP and GFP alone (as a control) in onion epidermal cells and foxtail millet root protoplasts. The constructs used were shown in Figure 2A. When observed by confocal microscopy, the green fluorescent protein (GFP) fluorescence of SiLEA14-GFP was distributed throughout the cell, consistent with the GFP control, both in the onion cells (Figure 2B) and the foxtail millet root protoplasts (Figure 2C). These results indicated that SiLEA14 was localized in the cytosol.

### *SiLEA14* expression profiles under normal and stress conditions

**Figure 3:**
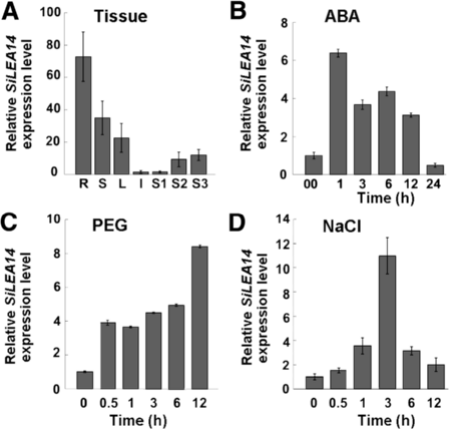
***SiLEA14*** **expression patterns in foxtail millet determined by qRT-PCR. (A)** Tissue-specific expression of *SiLEA14*. Total RNA was extracted from roots (R), stems (S), leaves (L), inflorescences (I) and seeds at 5 (S1), 15 (S2) and 25 (S3) days after pollination, respectively. Foxtail millet *Actin7* was used as an internal control. **(B-D)** *SiLEA14* transcripts accumulation in response to various treatments. Two-week-old foxtail millet seedlings were treated with 100 μM ABA **(B)**, 20% (v/v) PEG 6000 **(C)** and 250 mM NaCl **(D)** for the indicated times, respectively. Total RNA was extracted from the above-ground parts of ten plants at the indicated times after the treatments. Data represent means and standard errors for three biological replicates.

Quantitative reverse-transcription PCR (qRT-PCR) was carried out to reveal the temporal and spatial expression of *SiLEA14* in foxtail millet (Figure 3A). *SiLEA14* was expressed at the highest level in roots, followed by stems and leaves, and at the lowest level in inflorescences. After pollination, *SiLEA14* transcription was gradually upregulated with seed maturation, which indicated a potential role for *SiLEA14* in the maturation and desiccation phases of foxtail millet seed development.

The expression level of *SiLEA14* was also examined by qRT-PCR under various stresses. Under ABA treatment, *SiLEA14* transcription abruptly reached its highest level (six-fold) after 1 h treatment, and then decreased gradually and reverted almost to the control level at 24 h (Figure 3B). Under PEG stress, *SiLEA14* transcription was rapidly induced within 0.5 h and increased to a peak level (8-fold) at 12 h (Figure 3C). Salt treatment resulted in increased accumulation of *SiLEA14* to a maximum level (11-fold) only after 3 h (Figure 3D). These results indicated that SiLEA14 might play important roles in the responses to salt and drought stresses.

### SiLEA14 enhances salt tolerance in transformed *E. coli*

**Figure 4:**
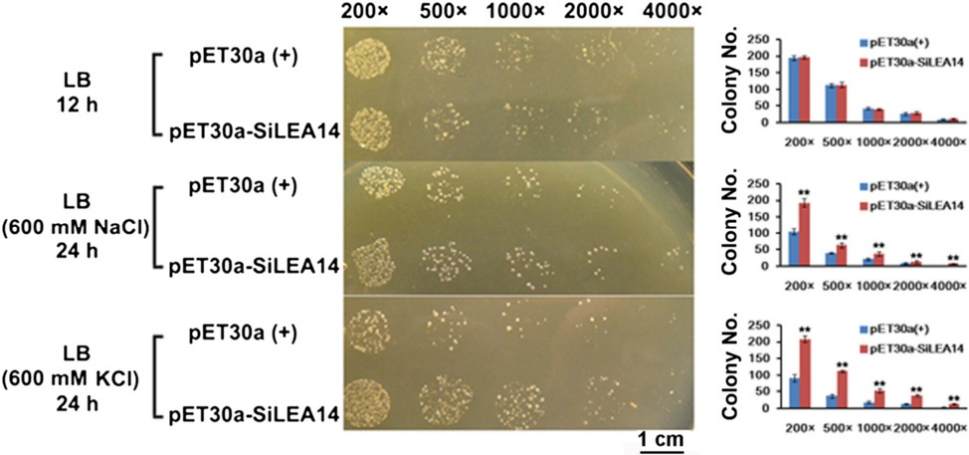
**Assay for salt stress tolerance of SiLEA14 transformed** ***E. coli*** **.** Transformant *E. coli* (BL21) with pET30a-SiLEA14 were grown on LB plates with or without 600 mM NaCl and KCl for 12 and 24 h, respectively. Transformant with empty vector pET30a(+) was used as a control. The number of colonies on the plate was calculated. The 200×, 500×, 1000×, 2000× and 4000× represent the dilution fold. Bar = 1 cm. Data represent means and standard errors for three biological replicates. Statistical significance was determined by Student’s *t*-test. **P < 0.01.

In both prokaryotes and eukaryotes LEA proteins might have similar protective mechanisms [28]. Therefore, the up-regulation of *SiLEA14* in response to NaCl prompted us to evaluate the salt stress tolerance of recombinant *E. coli* overexpressing *SiLEA14* (Figure 4). There are no significant differences in colony number between transformed *E. coli* harboring *SiLEA14* and the control under normal conditions, indicating that overexpression of *SiLEA14* did not affect the growth of *E. coli* recombinants in non-stress conditions. However, when grown on Luria–Bertani (LB) plates supplemented with 600 mM NaCl, the number of transformed cells was much higher than that of the control. A similar result was obtained on LB medium supplemented with 600 mM KCl. These results demonstrated that overexpression of *SiLEA14* in *E. coli* significantly enhanced tolerance to salt stress.

### SiLEA14 improves the abiotic stress resistance of transgenic Arabidopsis

**Figure 5:**
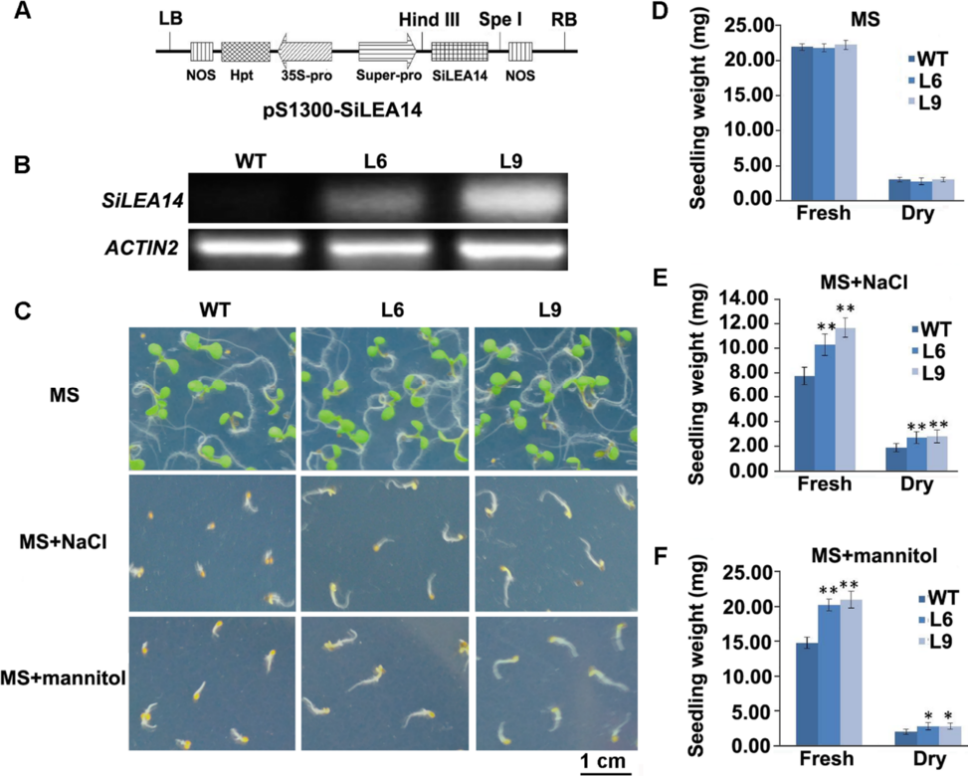
**Assay for salinity and mannitol stress tolerance of** ***SiLEA14*** **overexpression Arabidopsis. (A)** Diagram of the T-DNA region of the binary vector pS1300-SiLEA14. LB, left border; RB, right border; 35S-Pro, *cauliflower mosaic virus* 35S promoter; Hpt, hygromycin B phosphotransferase gene; Super-pro, Super promoter; SiLEA14, SiLEA14 gene; NOS, nopaline synthase terminator. **(B)** RT-PCR analysis of *SiLEA14* in transgenic Arabidopsis lines (L6 and L9). WT was used as a control. *Actin2* was a loading control. **(C)** The phenotype of transgenic and WT Arabidopsis germinated on MS, MS + 125 mM NaCl and MS + 250 mM mannitol, respectively, for 7 days. This experiment had three replicates, and each experiment comprised 30–40 plants. Bar = 1 cm. **(D-F)** Fresh/dry weights of seedlings germinated on MS, MS + 125 mM NaCl and MS + 250 mM mannitol, respectively. Data represent means and standard errors for three biological replications. Statistical significance was determined by Student’s *t*-test. *P < 0.05; **P < 0.01.

To assess the function of SiLEA14 in plants, it was overexpressed in Arabidopsis under the control of Super promoter (Figure 5A). At least 30 transgenic plants were obtained, and two independent homozygous T_3_ transgenic lines (L6 than L9) with high expression levels of *SiLEA14* (Figure 5B) were chosen for further investigation.

Under non-stress conditions, no significant differences in the fresh and dry weights were observed between the WT and transgenic plants (Figure 5C and D). However, when grown on Murashige and Skoog (MS) medium supplemented with 125 mM NaCl, the transgenic seedlings showed significantly larger cotyledons and longer roots than those of the WT, even though growth of these organs of the transgenic and WT plants was inhibited compared with normal conditions (Figure 5C). Consistently, the fresh and dry weights of transgenic seedlings were significantly higher than that of the WT (Figure 5E). Similar results were obtained when seedlings were treated with 250 mM mannitol (Figure 5C and F). These results suggest that SiLEA14 may enhance the osmotic stress resistance of transgenic Arabidopsis.

### Overexpression of *SiLEA14* increases the salt tolerance of transgenic foxtail millet

**Figure 6:**
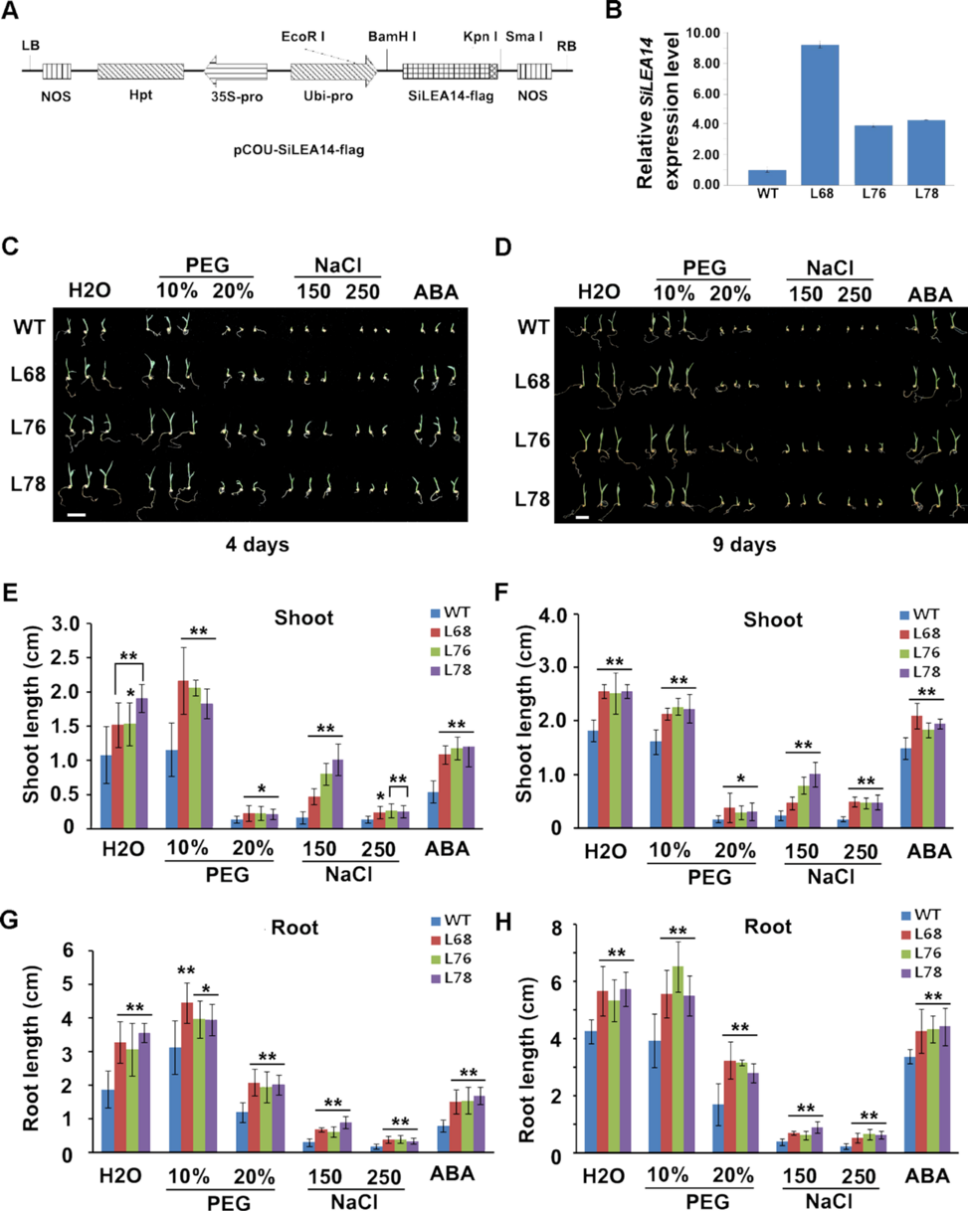
**Assay for NaCl, PEG and ABA stress on** ***SiLEA14*** **overexpression foxtail millet germination. (A)** Diagram of the T-DNA region of the binary vector pCOU-SiLEA14-flag. LB, left border; RB, right border; 35S-Pro, *cauliflower mosaic virus* 35S promoter; Hpt, hygromycin B phosphotransferase gene; Ubi-pro, maize Ubiquitin promoter; SiLEA14-flag, SiLEA14 gene fused with a flag tag; NOS, nopaline synthase terminator. **(B)** QRT-PCR analysis of *SiLEA14* in transgenic foxtail millet lines (L68, L76 and L78). *Actin7* was used as an internal control. **(C-D)** The phenotype of transgenic and WT foxtail millet under various abiotic stress treatment during the germination stage. The T_2_ seeds soaked in water (as control) or in water containing 150 mM NaCl, 250 mM NaCl, 10% PEG, 20% PEG and 10 μM ABA for 1 day at 30°C and then placed on the filter paper in a Petri dish wet with the same solutions mentioned above for 4 and 9 days, respectively. This experiment had three replicates, and each experiment comprised 30–40 plants. Bar = 1 cm. **(E-F)** The shoot length of transgenic and WT foxtail millet germinated under above conditions for 4 and 9 days, respectively. **(G-H)** The root length of transgenic and WT foxtail millet germinated under above conditions for 4 and 9 days, respectively. Data in **B**, **E**-**H** represent means and standard errors for three biological replicates. Statistical significance was determined by Student’s *t*-test. *P < 0.05; **P < 0.01.

To further analyze the function of *SiLEA14*, it was transformed into foxtail millet under the control of ubiquitin promoter (Figure 6A). The *SiLEA14* integration was confirmed by genomic PCR using two pairs of primer sets specific to *Hpt* and 35S promoter, respectively (Additional file 4). QRT-PCR analysis showed higher expression level of *SiLEA14* in transgenic lines L68, L76, and L78, which showed a 3:1 segregation ratio at T_1_ generation, than that of the WT (Figure 6B). The T_2_ generation of these three lines were used for further analysis.

First, we examined the salt tolerance of transgenic foxtail millet during germination, and the results were shown in Figure 6. Compared with the WT plants, transgenic lines showed better growth performance (Figure 6C) with enhanced shoot and root growth (Figure 6E and G) when germinated in water for 4 days. Under salt stress for 4 days, the transgenic lines showed significantly longer shoot and root than the WT (Figure 6E and G), even though the growth of both WT and transgenic lines were seriously suppressed (Figure 6C). Similar results were obtained for 9 days salt stress treatment (Figure 6D, F and H).

**Figure 7:**
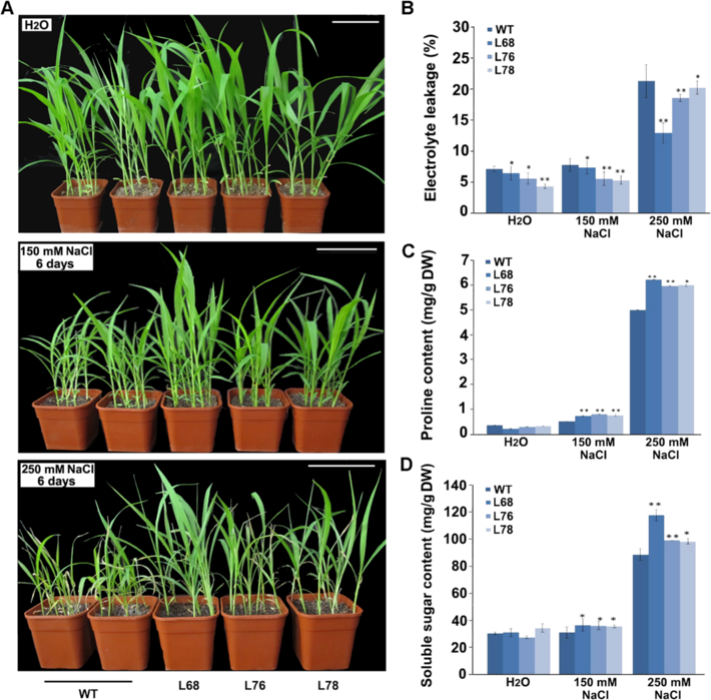
**Salt tolerance of** ***SiLEA14*** **overexpressing foxtail millet seedling. (A)** The phenotype of transgenic and WT foxtail millet under normal and salt stress condition. Two-week-old seedlings in soil were irrigated with water, 150 and 250 mM NaCl solution every 3 days. After 6 days, the phenotypes of the transgenic lines and WT were investigated. This experiment had three replicates; and six to eight plants grown in one plot were used in each experiment. Bar = 10 cm. **(B)** Relative electrolyte leakage in WT and transgenic lines after salt stress. **(C)** Proline content in WT and transgenic foxtail millet after salt stress. **(D)** Soluble sugar content in WT and transgenic foxtail millet after salt stress. Data in **B**-**D** represent means and standard errors for three biological replicates. Statistical significance was determined by Student’s *t*-test. *P < 0.05; **P < 0.01.

Further, we examined the salt tolerance of transgenic foxtail millet seedlings in soil. No striking differences in the height were observed between the transgenic and the WT plants under non-stress conditions, but wider leaves were observed in the transgenic foxtail millet compared with those of the WT (Figure 7A). This finding is in accordance with the result that transgenic lines showed longer shoot and root lengths when germinated in water compared with the WT (Figure 6C–H). Under 150 mM NaCl stress for 6 days, the first and second leaves of WT plants became curled, whereas there was no obvious effect on the transgenic lines. The WT plants were shorter than those of the transgenic lines. Under 250 mM NaCl stress for 6 days, the growth of WT plants was severely inhibited and leaves were curled, bleached, and senescent. However, the transgenic lines showed healthy growth except that only a few old leaves became bleached (Figure 7A).

Electrolyte leakage always occurs following membrane damage under salinity stress [29]. Furthermore, plants accumulate several metabolites, such as amino acids (e.g., proline), sugars, and sugar alcohols (e.g., mannitol and trehalose), to prevent detrimental changes caused by severe osmotic stress [30,31]. Therefore, we measured the electrolyte leakage and change in free proline and soluble sugar contents in control and *SiLEA14* transgenic lines with or without salinity stress, respectively. Under optimal or salt-stress conditions, the amount of electrolyte leakage in the transgenic lines was significantly lower than that of the WT plants (Figure 7B). This finding is in agreement with the result that the *SiLEA14* transgenic lines showed enhanced germination and growth compared with the WT under both normal and salt-stress conditions (Figures 6C–H, 7A). No significant differences in free proline and soluble sugar contents were observed between the WT and the transgenic lines under normal conditions. Under 150 and 250 mM NaCl stress, the free proline and soluble sugar contents increased in all plants. However, this increase was significantly more pronounced in the transgenic lines than in the WT plants (Figure 7C, D). All of these increased osmotic protectants are beneficial for protection of the plants against salt stress. Moreover, we noted that the transgenic line L68 demonstrated better salt tolerance than the other two transgenic lines (L76 and L78), which correlated with the higher accumulation of *SiLEA14* transcripts in L68. All of these results indicated that *SiLEA14* overexpressing foxtail millet is more tolerant to salt stress compared with the WT.

### Overexpression of *SiLEA14* increases drought resistance of transgenic foxtail millet

We also examined the drought resistance of transgenic foxtail millet during germination. When germinated in water for 4 days, the transgenic lines showed better growth performance than WT as described above (Figure 6C, E and G). Compared with in water, no obvious suppression under 10% PEG stress but serious suppression under 20% PEG stress for 4 days were observed (Figure 6C). The shoot and root length of transgenic lines was longer than that of the WT no matter germinated in water, 10% or 20% PEG (Figure 6E and G). When germinated in water or PEG for 9 days, similar results were obtained (Figure 6D, F and H).

**Figure 8:**
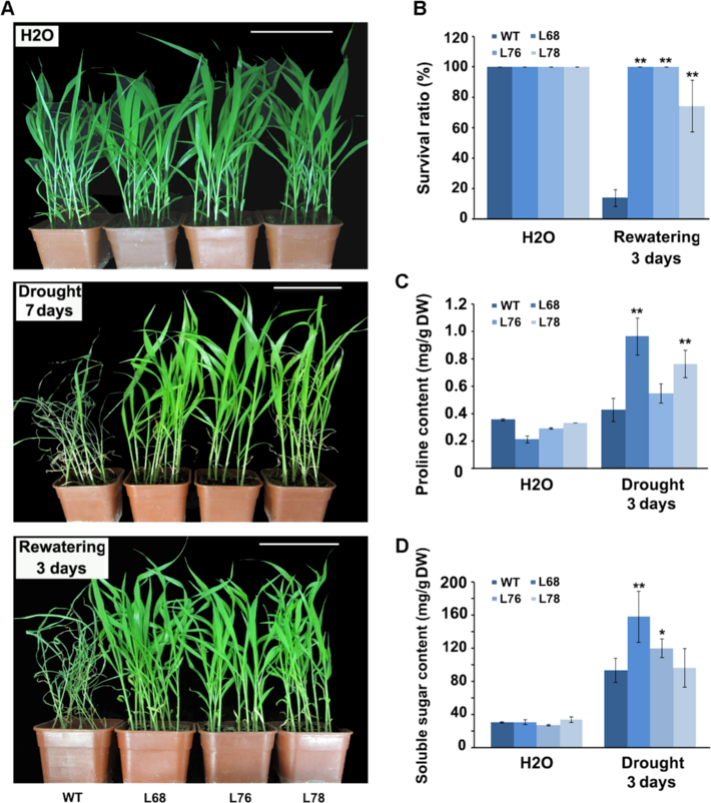
**Drought tolerance of** ***SiLEA14*** **overexpression foxtail millet in soil. (A)** The phenotypes of WT and transgenic foxtail millet under normal condition, drought stress for 7 days and after rewatering for 3 days. Two-week-old seedlings were used. Six to ten plants grown in one plot were used in each experiment. This experiment had three replicates. Bar = 10 cm. **(B)** The survival rate of WT and transgenic foxtail millet plants in **(A)** under drought stress. **(C)** Free proline content in WT and transgenic plants after drought stress. **(D)** Soluble sugar content in WT and transgenic plants after drought stress. Data in **B**-**D** represent means and standard errors for three biological replicates. Statistical significance was determined by Student’s *t*-test. *P < 0.05; **P < 0.01.

The observation that *SiLEA14* improved osmotic stress resistance during germination prompted us to investigate the drought resistance of transgenic foxtail millet seedlings in soil. As it is shown in Figure 8A, no significant differences in the height were observed between the transgenic lines and the WT when watered well, except that the leaves of the transgenic lines were wider. However, under drought stress for 7 days, more than 90% of WT leaves, but less than 20% of transgenic plant lines leaves, became curled and wilted. After rewatering, 74–100% plants of trangenic lines survived in contrast to only 14% of WT (Figure 8B).

The change in free proline and soluble sugar contents in control and *SiLEA14* transgenic lines with or without drought stress was examined. No significant differences in free proline and soluble sugar contents were observed between the WT and the transgenic lines under non-stress conditions. Under drought stress for 3 days, the free proline and soluble sugar contents increased in all WT and transgenic plants. However, a significantly higher increase in free proline content was observed in the transgenic lines L68 and L78 when compared to the WT. In addition, compared with the WT, the soluble sugar content increased significantly in the transgenic lines L68 and L76 (Figure 8C, D). Taken together these results indicate that *SiLEA14* overexpressing foxtail millet showed improved drought resistance.

### GUS activity is induced by various stresses in proSiLEA14::GUS transgenic *Arabidopsis*

**Figure 9:**
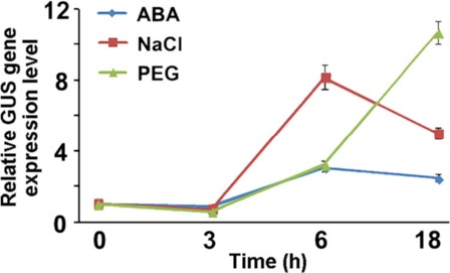
**Expression of GUS gene in pro** ***SiLEA14*** **::GUS transgenic Arabidopsis upon various stresses determined by qRT-PCR.** One-week-old seedlings were treated with 100 μM ABA, 250 mM NaCl and 20% (m/v) PEG 6000 solution for 0, 3, 6 and 18 h, respectively. Total RNA was extracted from at least 30 whole seedlings per treatment. The expression of GUS in proSiLEA14::GUS transgenic Arabidopsis was determined by qRT-PCR using GUS-specific primers (Additional file 4). *Actin2* was used to normalize the gene expression. Data represent means and standard errors for three biological replicates.

The putative *SiLEA14* promoter region was isolated from the foxtail millet genome using PCR. This fragment consisted in nt −1273 to +96 (transcription start site is +1) upstream of *SiLEA14* coding sequence. The *SiLEA14* promoter was fused to the *GUS* gene and transformed into Arabidopsis. Then, qRT-PCR was carried out to examine the GUS expression level under various stresses. As it is shown in Figure 9, GUS transcription was up-regulated to its highest level (about 3- and 8-fold increases) after 6 h treatment with ABA and NaCl, respectively. For PEG stress, GUS expression gradually increased and peaked (an almost 12-fold increase) after 18 h treatment. To further confirm these results, a histochemical GUS-staining assay was performed. No visible GUS activity was noted for the control. However, induction of GUS activity was observed with ABA, PEG or NaCl application for 3 to 18 h, although the GUS signals were not particularly strong (Additional file 5). Nevertheless, collectively these results suggest that the selected *SiLEA14* promoter sequence is enough to regulate ABA, PEG and NaCl induction of the gene.

### *SiLEA14* promoter contains stress-associated *cis*-elements

To characterize the mechanism of S*iLEA14* function, a 1273 bp S*iLEA14* promoter fragment (without the 96 bp 5′UTR) was subjected to a putative *cis*-acting regulatory element search using the Plant Cis-acting Regulatory DNA Elements (PLACE) database [32]. *Cis*-acting elements reported to be involved in ABA- and dehydration-mediated gene expression were identified (Table 1). Abscisic acid-responsive elements (ABREs) are common motifs for ABA- and dehydration-mediated regulation of transcription [33-35]. In the *SiLEA14* promoter, six ABRE-like sequences and four ABA- and dehydration-responsive ACGT-box motifs [34,36] were identified. A dehydration-responsive element (DRE)-like site, as a *cis*-acting element involved in ABA induction, drought, salinity, or cold stresses [37,38], was also identified at position −158. MYC and MYB binding sites have been identified as regulatory elements associated with ABA, drought, or cold responsiveness in plants [34,39-43]. In the SiLEA14 promoter, as many as 10 and three MYC- and MYB-like sequences, respectively, were observed. These results suggest that SiLEA14 may be under the regulation of DREB-, DBF-, CBF-, MYC- or MYB-like transcription factors as in Arabidopsis and rice. In addition, other regulatory *cis*-acting elements were identified in the *SiLEA14* promoter, such as low temperature-responsive, pathogen-, defense- and wounding-associated elements. However, whether SiLEA14 participates in these abiotic or biotic stresses awaits further investigation.

**Table 1:** **Putative** ***cis-*** **acting regulatory elements associated with ABA and various stresses in** ***SiLEA14*** **promoter region**

***Cis*** **-element name**	**Sequence**	**Position**	**Function**	**Ref.**
ABRE	ACGTG	−793, −792, −133, −78, −77	ABA-mediated regulation of transcription; Required for etiolation-induced expression of erd1 in Arabidopsis	[34,35]
	RYACGTGGY	−79	ABRE element involved in Arabidopsis dehydration-responsive gene rd22	[33]
	ACGT	−792, −382, −132, −77	Involved in ABA response; Required for etiolation-induced expression of erd1 in Arabidopsis	[34,36]
DRE	ACCGAC	−158	DBF1 and DBF2 binding site in the maize rab17 gene promoter involved in ABA induction	[38]
	RCCGAC	−158	DRE/CRT regulatory element involved in dehydration, cold or sanility responsiveness	[38]
	RYCGAC	−158	Binding site of barley CBF1 and CBF2 involved in cold acclimation	[37]
MYC	CACATG	−1034, −485	MYC binding site in rd22 gene of Arabidopsis; ABA-induction	[55]
	CANNTG	−1034, −867, −793, −666, −588, −553, −485, −468, −433, −78	MYC recognition site in rd22, CBF3 and many other genes in Arabidopsis; Binding site of ICE1 that regulates the transcription of CBF/DREB1 genes in the cold in Arabidopsis	[41,42]
	CATGTG	−1034, −485	MYC recognition sequence necessary for expression of erd1 in dehydrated Arabidopsis; Binding site of NAC which is stress-inducible	[34,43]
MYB	CNGTTR	−468, −465, −360	MYB binding site involved in regulation of genes that are responsive to water stress in Arabidopsis	[39,40]
	YAACKG	−468	MYB recognition sequence found in the promoters of rd22 and many other genes in Arabidopsis	[41]

M = C/A; Y = T/C; B = T/C/G; N = A/T/G/C; R = A/G; W = A/T; V = A/C/G; K = G/T.

## Discussion

Typical LEA proteins can retain water molecules and protect other proteins from aggregation or desiccation because of their highly hydrophilic properties [44]. Conversely, atypical LEA proteins have higher content of hydrophobic residues than typical LEAs. The latter have been speculated to be involved in diverse stress tolerances, although few studies have been carried out to characterize their functions [5,23,25]. Here, we reported the identification and characterization of SiLEA14, a novel atypical LEA member, in foxtail millet.

As a key phytohormone, ABA plays an important role in plant stress responses. Osmotic stress-regulated genes can be activated through both ABA-dependent and ABA-independent pathways [45]. However, it is considered that stress-signalling pathways for the activation of LEA-like genes completely independent of ABA may not exist [1]. In our study, *SiLEA14* accumulation was remarkably induced by ABA and peaked rapidly after 1 h treatment (Figure 3). In addition, *SiLEA14* promoter-driven GUS activity was distinctly stimulated by ABA (Figure 9). Compared with the WT, transgenic foxtail millet seeds showed better germination in ABA solution (Figure 6C–H). All of these results indicate that activation of SiLEA14 under salt and drought stresses may be dependent on ABA.

Yeast and *E. coli* heterologous systems have been widely used to investigate LEA gene functions [5,6,46]. In the present study, overexpression of *SiLEA14* protected *E. coli* cells from damage caused by salt stress (Figure 4). Overexpression of *SiLEA14* in Arabidopsis imparted increased tolerance to salt and mannitol stresses (Figure 5). This result suggests that SiLEA14 from foxtail millet, a monocot, can function properly in the dicot Arabidopsis. Interestingly, transgenic foxtail millet overexpressing *SiLEA14* exhibited superior germination and subsequent growth in soil compared with the WT even under normal conditions. Under salt and drought stresses, these differences were more remarkable, which were indicative of the key roles of SiLEA14 in foxtail millet (Figures 6, 7 and 8). Consistent with these findings, both *SiLEA14* transcription and *SiLEA14* promoter-driven GUS activity were remarkably induced by NaCl and PEG (Figures 3, 9 and Additional file 5). However, it should be noted that some discrepancies were observed between the expression pattern of the endogenous *SiLEA14* upon abiotic stress and ABA treatment (Figure 3) and the *SiLEA14* promoter-driven GUS transcript accumulation responsive to abiotic stress and ABA driven by *SiLEA14* promoter region in transgenic Arabidopsis (Figure 9). For example, the *SiLEA14* transcripts accumulated to the highest level after 250 mM NaCl treatment for 3 h (Figure 3), whereas the GUS transcripts reached the peak after 6 h treatment (Figure 9). Additionally, the histochemical analysis showed strong GUS signals in the petioles of transgenic Arabidopsis (Additional file 5), whereas the endogenous *SiLEA14* was highly expressed in roots (Figure 3). This is probably due to that *SiLEA14* is from the monocot foxtail millet and the GUS expression driven by *SiLEA14* promoter is performed in the dicot Arabidopsis.

So far, there is only one report on the functional mechanism of the atypical LEA protein in plants [25]. Overexpression of *IbLEA14*, a homologous gene of *SiLEA14*, enhanced tolerance to drought and salt stress in the sweetpotato calli. The contents of lignin in the *IbLEA14*-overexpressing calli were increased under normal conditions. The authors inferred that *IbLEA14* may be involved in these functions as a consequence of regulating increased lignin production. In our study, we found that overexpression of *SiLEA14* in Arabidopsis and foxtail millet obviously improved the osmotic stress resistance of transgenic plants. Meanwhile, the proline and sugar accumulated at a higher level in transgenic lines, especially in L68 which accumulated higher SiLEA14 transcripts, than WT after osmotic and NaCl stress. These results implied that overexpression of *SiLEA14* might up-regulate these metabolites. However, further study is needed.

The identification of *cis*-acting elements in the *SiLEA14* promoter may help to provide insight into the molecular mechanism of *SiLEA14* function. As a major *cis*-acting element, ABRE has been identified in the promoters of many ABA-inducible genes of plants such as the cotton LEA gene *D-113* [47]. The bZIP transcription factors AREB/ABF can bind to ABRE and activate ABA-inducible gene expression [48-51]. In the present study, ten *cis*-elements containing the ABRE ACGT-core were identified in the −793 to −77 bp region of the *SiLEA14* promoter. The DRE element was first identified in the promoter of *rd29A*, a gene responsive to dehydration stress in Arabidopsis [52]. In plants, the AP2 transcription factors DREB/CBF specifically bind to the DRE element to regulate the expression of the downstream stress-responsive genes [53]. There are interactions between ABRE and DRE in Arabidopsis rd29A gene expression in response to dehydration and high-salinity stresses [54]. One DRE-like element was predicted in the *SiLEA14* promoter. In Arabidopsis, an MYC transcription factor, AtMYC2 and an MYB transcription factor, AtMYB2, have been shown to bind the *cis*-elements CANNTG and C/TAACNA/G, respectively, to regulate expression of the dehydration-responsive gene *RD22* [41,55]. Ten MYC and three MYB-like sequences were identified in the *SiLEA14* promoter. Proteins that bind to these elements remain to be isolated. It is necessary to further elucidate the detailed functions of these putative regulatory *cis*-elements. In addition, pathogen-related elements were also identified. However, whether SiLEA14 participates in biotic stress responses needs to be investigated.

## Conclusions

In conclusion, this study characterizes a novel atypical LEA gene *SiLEA14* from foxtail millet. *SiLEA14* is responsive to ABA, PEG and NaCl and the SiLEA14 is localized in the cytosol. SiLEA14 improves the salt tolerance of *E. coli* transformant and transgenic Arabidopsis. Furthermore, overexpression of *SiLEA14* significantly enhances the salt and drought tolerances of transgenic foxtail millet. *SiLEA14* plays important roles in plant abiotic stress resistance and could be used in crops genetic engineering with the aim of improving stress tolerance.

## Methods

### Plant materials and growth conditions

Seeds of foxtail millet (*Setaria italica* cv. Jigu11), kindly provided by Prof. Xianmin Diao of the Institute of Crop Science, Chinese Academy of Agricultural Sciences, China, were geminated on moist gauze for 24 h at 30°C, and then grown in pots filled with nutrient soil and vermiculite mixed at 1:1 (v/v) in the controlled chamber (25–26°C, humidity 60-70%, under 16-h light/8-h darkness). Foxtail millet leaves, stems, roots, inflorescences and seeds at 5, 15 and 25 days after pollination were harvested and stored at −80°C after frozen in liquid nitrogen.

Seeds of *Arabidopsis thaliana* (Col-0), after stratification, were plated on ½MS medium with 2% sucrose and 0.7% agar for three weeks at 21–22°C with a 16 h/8 h (day/night) photoperiod and 60-70% relative humidity. Then, the young seedlings were planted on fertilized soil and grown in the same conditions.

### Stress treatments

For *SiLEA14* expression profile in response to ABA, NaCl and PEG, two-week-old foxtail millet seedlings were carefully removed from soil and washed. The cleaned plants were fixed in plastic foam, and grown hydroponically in water for 1 d. Then, the seedling roots were immersed separately in water containing 100 μM ABA, 250 mM NaCl and 20% (m/v) PEG 6000 for the indicated time in Figure 3, respectively. Six seedlings were used in each treatment. After drying on the filter paper, the seedlings were harvested, and then immediately frozen in liquid nitrogen and stored at −80°C until use.

For GUS expression in response to ABA, NaCl and PEG, about 20 one-week-old proSiLEA14::GUS transgenic Arabidopsis plants were carefully removed from the plates and immersed in water containing 100 μM ABA, 250 mM NaCl and 20% (m/v) PEG 6000 for 0, 3, 6 and 18 h, respectively. For histochemical GUS staining, three-week-old proSiLEA14::GUS transgenic Arabidopsis plants were used.

### Cloning and sequence analysis of *SiLEA14*

The full-length *SiLEA14* was amplified by RACE in accordance with the manufacturer’s protocol (GeneRacer™ Kit, Invitrogen, Carlsbad, CA, USA). The products were cloned into the pMD19-T vector (Takara, Shiga, Japan) and sequenced. Primer sets used are listed in Additional file 4. The isoelectric point and molecular mass predictions were estimated using the compute pI/Mw tool (http://expasy.org/tools/pi_tool.html). Analysis of protein hydropathy was done by constructing hydropathy plots with the Kyte and Doolittle algorithm (http://ipsort.hgc.jp/) [56]. Motif analysis was performed using the Pfam program (http://www.ebi.ac.uk/Tools/InterProScan/). The grand average of hydropathy (GRAVY) and instability index of deduced proteins were predicted using the ProtParam program (http://au.expasy.org/tools/protparam.html). Sequence similarities were determined using the BLAST program and the GenBank database on the NCBI web server. The complete amino acid sequences of subgroup 5C LEA proteins were used to construct a phylogenetic tree. Sequence alignment was performed with ClustalW and adjusted manually. A phylogenetic tree was constructed with the neighbor-joining method using the MEGA4.0 program [57]. Sequence logos for subgroup 5C LEA14 were obtained with the WebLogo website http://weblogo.berkeley.edu/logo.cgi [58].

### RNA extraction, semi-quantitative PCR and qRT-PCR

Total RNA was extracted using TRIzol reagent (Invitrogen, Carlsbad, CA, USA) and first-strand cDNA was prepared with SuperScript III Reverse Transcriptase (Invitrogen, Carlsbad, CA, USA) after digestion with RNase-free DNase I (Takara, Shiga, Japan). The semi-quantitative RT-PCR were conducted as follows: 95°C for 3 min, then 25 cycles of 95°C for 30 s, 54°C for 30 s, and 72°C for 30 s for both *SiLEA14* and *Actin2*. For qRT-PCR, 100 ng of cDNAs were used as template in a 20 μL reaction system, containing 10 μL 2× SYBR Premix Ex Taq II (TaKaRa, Shiga, Japan), and 0.5 μM each specific forward and reverse primer (Additional file 4). Amplification was performed using the Bio-Rad CFX96 Real-Time PCR System C1000 Thermal Cycler (Bio-Rad, USA) as follows: 95°C for 30 s, 35 cycles of 95°C for 10 s, 60°C for 10 s, and 72°C for 10 s. Arabidopsis *Actin2* (accession number: NM_180280) and foxtail millet *actin7* (accession number: NM_001280818) were used as the endogenous references. Primers used were listed in Additional file 4.

### Subcellular localization of SiLEA14

The coding sequence of *SiLEA14* without the terminating codon was amplified and inserted into the *Xba*I/*Sma*I sites of pROK219-GFP to generate the construct pROK219-SiLEA14-GFP. Onion epidermal cells were bombarded with the constructs pROK219-GFP and pROK219-SiLEA14-GFP, which were validated by sequencing, using a particle gun-mediated system PDS-1000/He (Bio-Rad, Hercules, CA, USA). Foxtail millet protoplast isolation and transfection were carried out according to the procedure described by Zhai et al. [59]. Root tissues from 7-day-old seedlings were sliced and then incubated in a solution containing 1.5% Cellulase RS, 0.75% Macerozyme R10, 0.6 M mannitol, 10 mM MES, 0.1% BSA and 1 mM CaCl_2_ for 4–5 h at 28°C in the dark with gentle swirling (50 rpm). The constructs pROK219-SiLEA14-GFP and pROK219-GFP were incubated with protoplasts and 40% PEG 4000 for 20 min at room temperature for transient transformation, respectively. GFP signals were observed with a confocal laser scanning microscopy (LSM 510, Carl Zeiss MicroImaging GmbH, Jena, Germany).

### Assay for salt-stress tolerance of *E. coli* transformants

The coding sequence of SiLEA14 without the stop codon was amplified and cloned into the *Eco*RV/*Xho*I sites of pET30a(+) to construct the expression vector pET30a-SiLEA14, which was then transformed into *E. coli* host strain BL21. The pET30a(+) empty vector was used as the control. The expression of SiLEA14 in the recombinant cells was confirmed by SDS-PAGE analysis (Additional file 6). Transformed *E. coli* BL21 cells carrying pET30a-SiLEA14 or pET-30a (+) were grown in LB liquid medium supplemented with 100 μg/ml ampicillin overnight at 37°C, respectively. The bacterial cultures were diluted 100-fold using fresh liquid LB, and allowed to incubate for 2–3 h at 37°C until OD_600_ = 0.5–0.6. Isopropylthio-β-D-galactoside was then added to the cultures to a final concentration of 1 mM, and the bacteria were cultured for a further 4 h at 30°C to induce expression of the inserted gene. All induced cultures were adjusted to OD_600_ = 0.6 using fresh liquid LB medium with 100 μg/ml ampicillin. To measure responses to salt stress, the samples were diluted by 200-, 500-, 1000-, 2000- and 4000-fold with fresh LB medium supplemented with 100 μg/ml ampicillin. Five microliters of each diluted sample were plated on LB agar plates, LB agar plates supplemented with 600 mM KCl and 600 mM NaCl, respectively. After incubation for 12 h on LB agar plates or 24 h on LB agar plates supplemented with 600 mM KCl and 600 mM NaCl at 37°C respectively, the numbers of colonies were calculated. Growth was measured at least three times.

### Generation of transgenic plants

For *SiLEA14* overexpression in Arabidopsis, the coding region of *SiLEA14* was amplified and ligated into the *Hin*dIII/*Spe*I sites of pSB1300, which was kindly provided by Prof. Shuhua Yang of the College of Biological Sciences, China Agricultural University, China, to generate the construct pSB1300-SiLEA14 in which SiLEA14 was controlled by Super promoter. For *SiLEA14* promoter assay, the putative *SiLEA14* promoter was isolated from the foxtail millet genome using PCR and cloned into the *Sal*I/*Eco*RI sites of pCAMBIA1391-GUS to generate the construct pCAMBIA1391-proSiLEA14-GUS. After validation by sequencing, the constructs were introduced into *Agrobacterium tumefaciens* strain GV1301, and transformed into Arabidopsis by the floral dip method [60]. Seeds were obtained following self-pollination.

For *SiLEA14* overexpression in foxtail millet, the coding region of *SiLEA14* fused with a flag tag at the 3′end was amplified and ligated into the *Sac*I/*Kpn*I sites of the binary vector pCOU [61], which includes the ubiquitin promoter to drive transgene expression. After confirmation by sequencing, the recombinant plasmid pCOU-SiLEA14-flag was introduced into *A. tumefaciens* strain LBA4404. Transgenic foxtail millet plants were obtained by *Agrobacterium*-mediated transformation as described previously [62,63]. Seeds were obtained following self-pollination.

### Abiotic stress-tolerance assay of *SiLEA14* transgenic Arabidopsis

Seeds of WT and T_3_ transgenic Arabidopsis plants were surface sterilized by the vapor-phase method [60], and sown on MS, MS + 125 mM NaCl and MS + 250 mM mannitol media, respectively. After 3 days vernalization at 4°C, they were cultured in a controlled chamber (22°C, humidity 40-50%, 120–150 μmol/m^−2^ s^−1^ under 16-h light/8-h darkness). Photographs were taken after 7 days culture. Fresh and dry weights of each sample were calculated based on the average weight of 20 individual plants.

### Abiotic stress tolerance assay of *SiLEA14* transgenic foxtail millet

For analysis in the germination stage, 30–40 seeds of both WT and T_2_ transgenic millet were geminated on the filter paper in a Petri dish wet with water (as control) or water containing 150 mM NaCl, 250 mM NaCl, 10% PEG, 20% PEG and10 μM ABA for 1 day at 30°C, respectively. Photographs were taken after 4 and 9 days, respectively. Shoot and root lengths were measured.

For salt tolerance assay in soil, two-week-old seedlings were irrigated with water, 150 and 250 mM NaCl solution every 3 days, respectively. After 6 days, the phenotypes of the transgenic lines and WT were investigated. Six to eight plants grown in one plot were used in each experiment. Two fully expanded young leaves from each foxtail millet plant per plot were harvested and cut into 1 cm segment for electrolyte leakage measurement.

For drought tolerance assay in soil, two-week-old seedlings were deprived of water for 7 days. Subsequently, the plants were irrigated with water and grown for 3 days. Six to ten plants grown in one plot were used in each experiment. The survived plants were counted. Above-ground parts of treated seedlings were collected and used to measure proline and soluble sugar contents.

### Electrolyte leakage assay

Leaf tissue (0.1 g) from each sample was washed and immersed in 20 mL deionized water with 150 rpm shaking for 16 h. The initial electrical conductivity (L1) of the sample was detected using a FE-30 conductivity meter (Mettler-Toledo, Columbus, OH, USA). Then, the samples were autoclaved at 121°C for 10 min and cooled to room temperature. The ultimate conductivity (L2, maximum conductivity of tissues) was measured. Relative electrical conductivity (L) was calculated as the ratio of L1/L2.

### Proline content measurement

Free proline content of foxtail millet plants was measured using the method described by Bates et al. [64]. Leaf tissue (0.1 g, dry weight) was used to extract free proline in 3% sulphosalicylic acid at 95°C for 15 min. Then, 2 mL of supernatant was transferred to a new tube and reacted with 2 mL acetic acid and 2 mL acidified ninhydrin reagent for 30 min at 95°C. Next, 5 ml of toluene was added to the tube with full shaking. The absorbance of the toluene layer was determined at 520 nm.

### Soluble sugar content measurement in foxtail millet leaves

Soluble sugar content of foxtail millet plants was examined using the method of Yemm and Willis [65]. Leaf tissue (0.1 g, dry weight) was used to extract soluble sugar in 7 ml of 80% ethanol with constant stirring at 80°C for 2 h. Ethanol was evaporated in the boiled water bath. Then, 1 ml water and 5 ml of 0.15% anthrone solution was added. After incubation at 95°C for 15 min and cooling to room temperature, the absorbance of the reaction solution was determined at 620 nm. Glucose was used as a standard.

### Histochemical GUS staining

Histochemical GUS staining was performed as described by Jefferson et al. [66]. After the GUS staining, Arabidopsis seedlings were treated with 70% ethanol to remove chlorophyll from the GUS-stained tissue.

### Statistical analysis

The survival rate, fresh/dry weights, relative electrolyte leakage rate, proline content and soluble sugar content data were subjected to Student’s t-test analyses using GraphPad Prism 5. All of the experiments were repeated three times.

### Availability of supporting data

The *SiLEA14* sequence was deposited in GenBank with an accession number of KJ767551. The data supporting the results of this article are included within the article and its additional files.

## Additional files

Additional file 1:
**Sequence analyses of** ***SiLEA14.*** (A) The nucleotide and deduced amino acid sequences of *SiLEA14*. The transcription site was indicated in large red case. The start codon and stop codon of *SiLEA14* are marked in normal red case. The regular distribution of polar amino acid residues in SiLEA14 protein was underlined in black for acidic and amide residuces (N, D, Q and E) and red lines for basic residues (R, K and H). (B) The gene structure of *SiLEA14*. The position and length of the exons and intron of the SiLEA14 gene are displayed schematically. The green rectangles indicate the exons, and the black line indicates the intron. (C) Hydropathy analysis of SiLEA14 protein sequence using Kyte-Doolittle algorithm. Amino acid position is plotted on the x axis beginning with the N-terminus. Hydrophobic regions (I, II, and III) in the conserved motif were marked. (D) Amino acid content (mol %), GRAVY and instability index of SiLEA14. Additional file 2:
**Sequence information of SiLEA14 homologs.**
Additional file 3:
**The sequence logo for the conserved domain of SiLEA14 protein and its homologs.**The overall height of each stack represents the conservation of the protein sequences at that amino acid position, whereas the height of letters within each stack indicates the relative frequency of the corresponding amino acid. Additional file 4:
**Primer sets used in this study.**
Additional file 5:
**Histochemical GUS staining of transgenic Arabidopsis containing pro** ***SiLEA14*** **-GUS fusion upon various stresses.** Three-week-old seedlings subjected to 100 μM ABA, 20% PEG or 250 mM NaCl for 0, 3, 6 and 18 hours, respectively, were used. Additional file 6:
**SiLEA14 expression in the recombinant** ***E. coli*** **cells by** **SDS-PAGE** **analysis.** M. Protein marker. Lane 1, pET30a (+) uninduced. Lane 2, pET30a (+) induced. Lane 3, pET30a-SiLEA14 uninduced. Lane 4, pET30a-SiLEA14 induced. Lane 5, Supernate of pET30a-SiLEA14 induced extracts. Lane 6, Pellet of pET30a-SiLEA14 induced extracts. Red triangle represents the HIS tag protein (7 KD). Green triangles represent the SiLEA14 fusion protein.
