# Supplementary figures and images for: SiLEA14, a novel atypical LEA protein, confers abiotic stress resistance in foxtail millet
Source: BMC Plant Biol. 2014 Nov 18;14:290. doi: 10.1186/s12870-014-0290-7 (PMC4243736; doi:10.1186/s12870-014-0290-7)

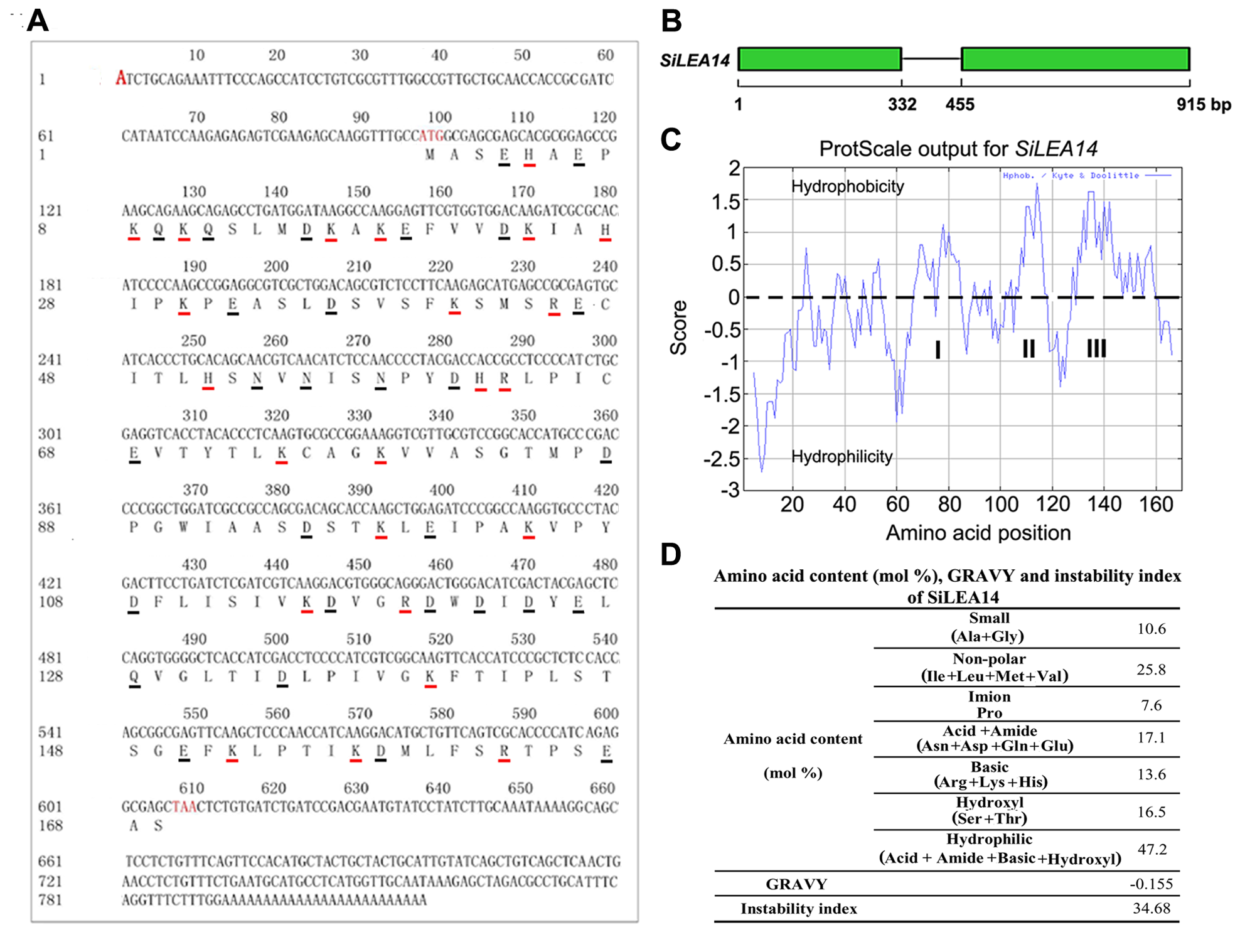

Supplement: Additional file 1: — Sequence analyses of SiLEA14. (A) The nucleotide and deduced amino acid sequences of SiLEA14. The transcription site was indicated in large red case. The start codon and stop codon of SiLEA14 are marked in normal red case. The regular distribution of polar amino acid residues in SiLEA14 protein was underlined in black for acidic and amide residuces (N, D, Q and E) and red lines for basic residues (R, K and H). (B) The gene structure of SiLEA14. The position and length of the exons and intron of the SiLEA14 gene are displayed schematically. The green rectangles indicate the exons, and the black line indicates the intron. (C) Hydropathy analysis of SiLEA14 protein sequence using Kyte-Doolittle algorithm. Amino acid position is plotted on the x axis beginning with the N-terminus. Hydrophobic regions (I, II, and III) in the conserved motif were marked. (D) Amino acid content (mol %), GRAVY and instability index of SiLEA14. [file 12870_2014_290_MOESM1_ESM.tiff]

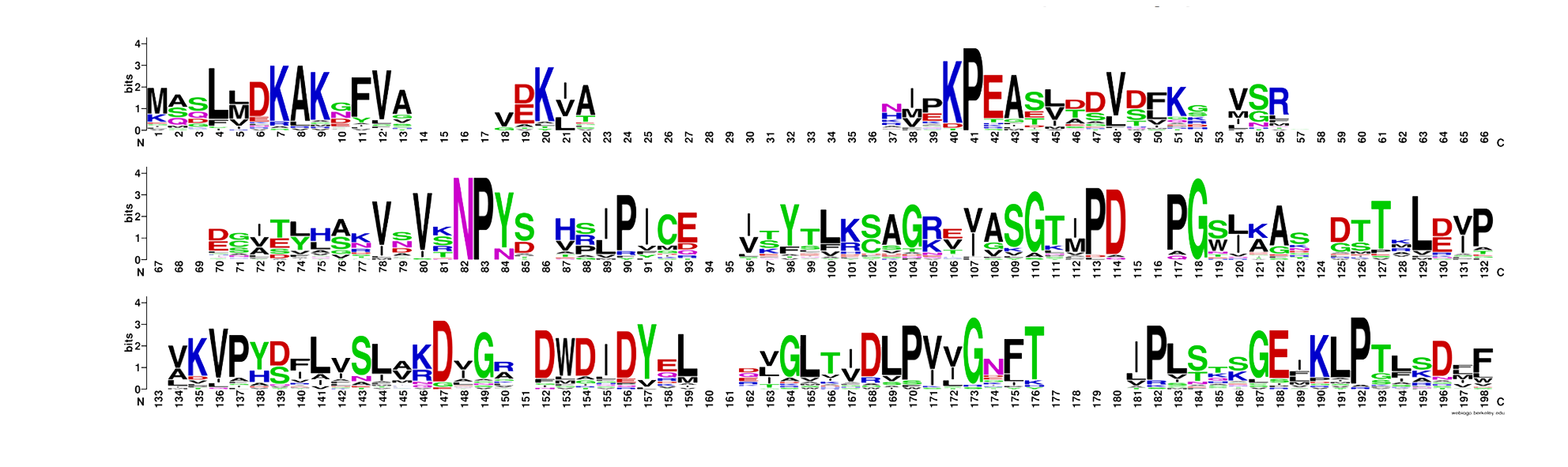

Supplement: Additional file 3: — The sequence logo for the conserved domain of SiLEA14 protein and its homologs.The overall height of each stack represents the conservation of the protein sequences at that amino acid position, whereas the height of letters within each stack indicates the relative frequency of the corresponding amino acid. [file 12870_2014_290_MOESM3_ESM.tiff]

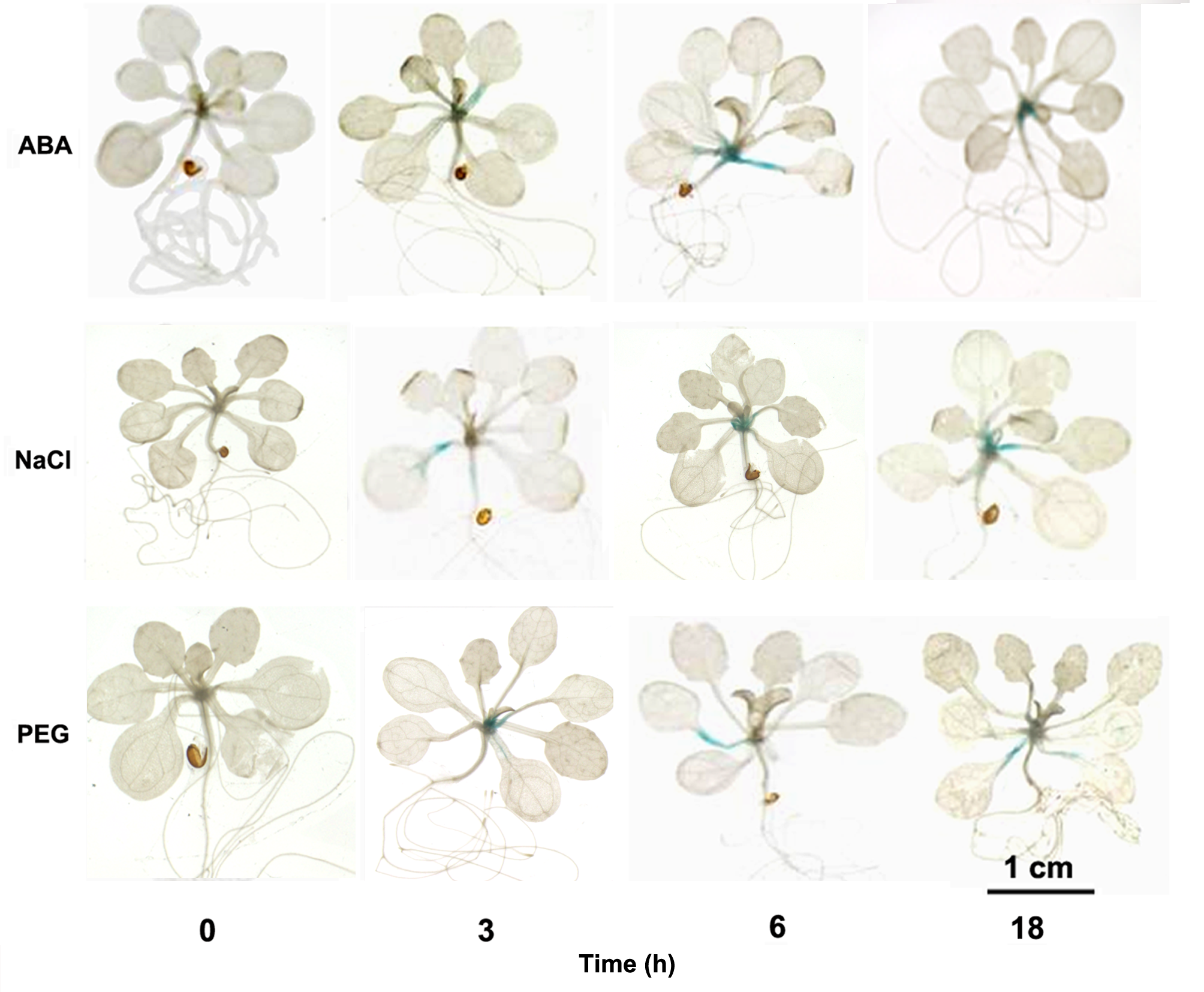

Supplement: Additional file 5: — Histochemical GUS staining of transgenic Arabidopsis containing pro SiLEA14 -GUS fusion upon various stresses. Three-week-old seedlings subjected to 100 μM ABA, 20% PEG or 250 mM NaCl for 0, 3, 6 and 18 hours, respectively, were used. [file 12870_2014_290_MOESM5_ESM.tiff]

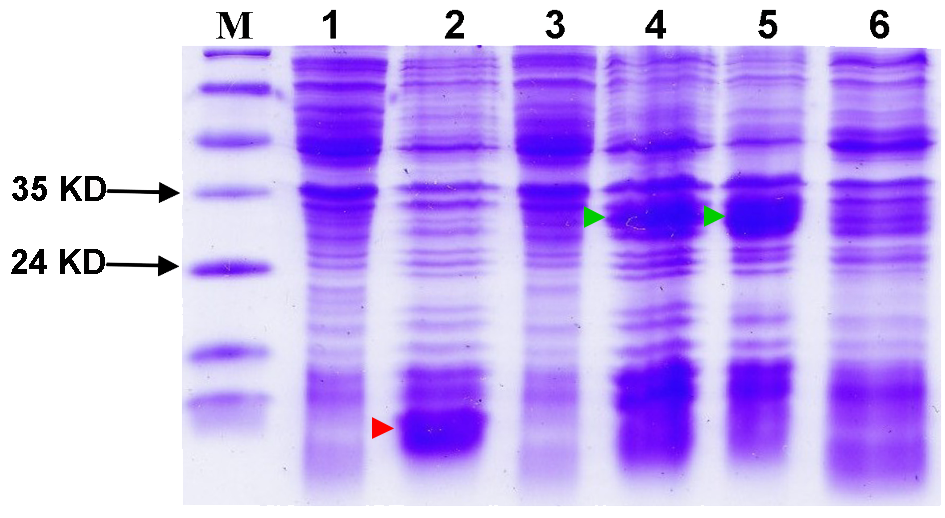

Supplement: Additional file 6: — SiLEA14 expression in the recombinant E. coli cells by SDS-PAGE analysis. M. Protein marker. Lane 1, pET30a (+) uninduced. Lane 2, pET30a (+) induced. Lane 3, pET30a-SiLEA14 uninduced. Lane 4, pET30a-SiLEA14 induced. Lane 5, Supernate of pET30a-SiLEA14 induced extracts. Lane 6, Pellet of pET30a-SiLEA14 induced extracts. Red triangle represents the HIS tag protein (7 KD). Green triangles represent the SiLEA14 fusion protein. [file 12870_2014_290_MOESM6_ESM.tiff]
