# Supplementary material for: SiLEA14, a novel atypical LEA protein, confers abiotic stress resistance in foxtail millet
Source: BMC Plant Biol. 2014 Nov 18;14:290. doi: 10.1186/s12870-014-0290-7 (PMC4243736; doi:10.1186/s12870-014-0290-7)
Supplement: Additional file 4: — Primer sets used in this study. [file 12870_2014_290_MOESM4_ESM.docx]

**Additional file 4.** Primers used in this study.

| **Gene** | **Use** | **Primer name** | **Primer sequences** |
| --- | --- | --- | --- |
| *SiLEA14* | *SiLEA14* full length cloning by RACE | 5'-Reverse GSP Primer | 5'-GATGTCCCAGTCCCTGCCCACGTCCTT-3' |
|  |  | 5'-Reverse GSP Nested Primer | 5'-GGCGGTGGTCGTAGGGGTTGGAGATGTT-3' |
|  |  | 3'-Reverse GSP Primer | 5'-CGGCACCATGCCCGACCCCGGCTGGAT-3' |
|  |  | 3'-Reverse GSP Nested Primer | 5'-CCATCCCGCTCTCCACCAGCGGCGAGTT-3' |
|  | ORF cloning for pROK219-SiLEA14-GFP construction | SiLEA14-*Xba* I | 5'-GCTCTAGAATGGCGAGCGAGCACG-3' |
|  |  | SiLEA14-*Sca* I | 5'-GAGTACTGCTCGCCTCTGATGGG-3' |
|  | ORF cloning for pCOU-SiLEA14-flag construction | SiLEA14-*Sac* I | 5'-CGAGCTCAAGATGACGATGAAGC-3' |
|  |  | SiLEA14-*Kpn* I | 5'-CGGTACCTTACTTGTCGTCGTCGTCCTTATAGTCGAAGCAGCGCGGGAC-3' |
|  | ORF cloning for pSB1300-SiLEA14 construction | SiLEA14-*Hind* III | 5'-CCCAAGCTTATGGCGAGCGAGCAC-3' |
|  |  | SiLEA14-*Spe* I | 5'-GACTAGTGCTCGCCTCTGATGGGG-3' |
|  | ORF cloning for pET30a-SiLEA14 construction and transgenic Arabidopsis PCR detection | SiLEA14- *Eco*R V | 5'- AATGGATATCATGGCGAGCGAGCACG-3' |
|  |  | SiLEA14- *Xho* I | 5'-CCGCTCGAGTGCTCGCCTCTGATGGGGTGC-3' |
|  | RT-PCR and qRT-PCR assay for *SiLEA14* in transgenic Arabidopsis and foxtail millet, respectively | qSiLEA14-3 | 5'- CTGATGGATAAGGCCAAGGA-3' |
|  |  | qSiLEA14-4 | 5'- TGTAGGTGACCTCGCAGATG-3' |
| *Hpt* | Transgenic foxtail millet PCR | hpt-3 | 5'-TCGGCTCCAACAATGTCCTG-3' |
|  |  | hpt-4 | 5'-CGGTCGGCATCTACTCTATTCC-3' |
| *35S promoter* |  | 35S-1 | 5'-CTAACAGAACTCGCCGTAAAGAC-3' |
|  |  | 35S-2 | 5'-GTGACAGATAGCTGGGCAATGGA-3' |
| *Foxtail millet actin7* | qRT-PCR | SiActin7-1 | 5'-GAACCCCAAGGCTAACAG-3' |
|  |  | SiActin7-2 | 5'-CAGTGGTGGTGAAGGAGTA-3' |
| *Arabidopsis Actin2* | qRT-PCR | AtActin2-1 | 5'- GGTAACATTGTGCTCAGTGGTGG-3' |
|  |  | AtActin2-2 | 5'- AACGACCTTAATCTTCATGCTGC-3' |
| SiLEA14 promoter | SiLEA14 Promoter cloning | pSiLEA-1 | 5'- ATCAAGGCAAGCAACGCATCTCA-3' |
|  |  | pSiLEA-2 | 5'- AAACCTTGCTCTTCGACTCTCTCTT-3' |
| GUS | qRT-PCR | GUS-1 | 5'- ATCAGCACGTTATCGAATCCTT-3' |
|  |  | GUS-2 | 5'- CCGGGTGAAGGTTATCTCTATG-3' |
| GUS-NOS | *SiLEA14* promoter::*gus* transgenic plants PCR | GUS-1 | 5’-CTGCGACGCTCACACCGATACC-3’ |
|  |  | NOS-2 | 5’-CTCAGTAGGATTCTGGTGTGTGCGC-3’ |
